# *Bacillus amyloliquefaciens* SC06 Protects Mice Against High-Fat Diet-Induced Obesity and Liver Injury via Regulating Host Metabolism and Gut Microbiota

**DOI:** 10.3389/fmicb.2019.01161

**Published:** 2019-05-28

**Authors:** Yang Wang, Yanping Wu, Baikui Wang, Han Xu, Xiaoqiang Mei, Xiaogang Xu, Xiaoping Zhang, Jiajia Ni, Weifen Li

**Affiliations:** ^1^Key Laboratory of Animal Feed and Nutrition of Zhejiang Province, Institute of Feed Science, College of Animal Sciences, Zhejiang University, Hangzhou, China; ^2^College of Animal Science and Technology, Qingdao Agricultural University, Qingdao, China; ^3^Key Laboratory of High Efficient Processing of Bamboo of Zhejiang Province, China National Bamboo Research Center, Hangzhou, China; ^4^Department of Hepatobiliary Surgery II, Guangdong Provincial Research Center of Artificial Organ and Tissue Engineering, Zhujiang Hospital of Southern Medical University, Guangzhou, China; ^5^State Key Laboratory of Organ Failure Research, Southern Medical University, Guangzhou, China

**Keywords:** *Bacillus amyloliquefaciens*, high-fat diet, liver injury, gut microbiota, oxidative stress

## Abstract

Obesity and the related liver diseases are prevalent around the world. Although probiotics have been shown to prevent obesity through multiple ways, only few researches investigated the lipid-lowering effects of probiotic *Bacillus*. Moreover, the limited results consistently suggested that *Bacillus* regulated genes related to lipogenesis and oxidation, but no further exploration was made. Our previous study revealed that *Bacillus amyloliquefaciens* SC06 has a potent antioxidant capacity *in vitro*. The aim of this study is to investigate the effects of SC06 on obesity and the associated liver injury of high-fat diet (HFD)-fed-mice and its underlying mechanism. By feeding normal chow (NC), NC+SC06, HFD, and HFD+SC06 to mice, we found that SC06 improved body weight gain, hepatic steatosis, and glucose metabolism of HFD-mice. Furthermore, SC06 also increased the antioxidant capacity of mice through Nrf2/Keap1 signaling pathway. High-throughput sequencing of 16S rRNA gene showed that HFD changed the gut microbiota dramatically, while HFD+SC06 decreased the ratio of Firmicutes/Bacteroidetes and increased *TM7* abundance. More differences were also found in lower taxa. Altogether, SC06 is a potential probiotic that decreases HFD-related lipid accumulation and liver injury via regulating the antioxidant capacity and host gut microbiota.

## Introduction

With more than 1.4 billion overweight or obese adults (Hall et al., 2015), obesity has become a major threat to public health in both developed and developing countries (Haslam and James, 2005). In the United States, more than 36% of adults are obese with a body mass index (BMI) greater than 30 kg/m^2^ (Ogden et al., 2014). The mean BMI in Chinese adults also increased from 22.7 kg/m^2^ in 2004 to 23.7 kg/m^2^ in 2010 with the prevalence of obesity increased from 3.3% in 2004 to 5.2% in 2010 (Jiang Y. et al., 2015). Therefore, it is now beginning to replace under-nutrition and infectious diseases as the most significant contributor to ill health (Dietz et al., 2015).

Obesity and obesity-related metabolic diseases are caused by multiple factors, including diet, gene, environment and mentality (Kopelman, 2000). High fat and sugar intake are the main causes of obesity (Tappy and Le, 2010). Although the mechanisms that underlie high-fat diet (HFD)-induced physiopathologic changes have yet to be elucidated, it is unveiled that HFD regulates the genes encoding enzymes and influences nutrient-sensing signal (Kim et al., 2004; Birse et al., 2010). Associated with obesity, non-alcoholic fatty liver disease (NAFLD) is the major reason for abnormal liver function worldwide (Li et al., 2014). Approximately 5% of NAFLD individuals develops hepatic inflammation and fibrosis (Barreyro et al., 2015). Obesity is also linked to oxidative stress (Furukawa et al., 2004; Charradi et al., 2013). Multiple possible sources of oxidant stress in the fatty liver may constitute the “second hit” for cellular injury in non-alcoholic steatohepatitis (Carmiel-Haggai et al., 2004). Therefore, it is believed that antioxidants may be effective agents in treating active steatohepatitis through attenuating the secondary injury. Foster et al. (2011) found that antioxidant atorvastatin combined with vitamin C and E was effective in reducing the hepatic steatosis in individuals with NAFLD. S-adenosylmethionine, which generates glutathione (GSH), also had the potential to treat NAFLD (Musso et al., 2013).

During the past decades, it became clear that gut microbiota is instrumental in the control of host energy metabolism. Germ-free mice had about 40% less total body fat than the “normal” mice (Backhed et al., 2004). The conventionalization contributed to a 60% increase in body fat of conventionalized germ-free mice with the gut microbiota from the “normal” mice. Besides, the interactions between liver and gut play a critical role in NAFLD onset and progression (Jumpertz et al., 2011). Although the involvement of gut microbiota in NAFLD pathogenesis is complex, several reports demonstrated that intestinal bacteria may cause NAFLD via modulating low-grade inflammation, energy homeostasis, choline metabolism, bile acid homeostasis and endogenous ethanol generation (Kirpich et al., 2015).

Probiotics are non-pathogenic bacteria that exert a beneficial influence on host health (Rajput et al., 2013). As the widely studied probiotics, *Lactobacillus* and *Bifidobacteria* can improve lipid metabolism through assimilating cholesterol, binding cholesterol of cellular surface and deconjugating bile (Kumar et al., 2012). Moreover, *Lactobacillus* consumption can also alter the gut microbiota community structure and enrich the operational taxonomic units that are negatively correlated with metabolic syndrome phenotypes, including NAFLD (Compare et al., 2012; Wang et al., 2015; Park et al., 2017). However, as far as we know, only few studies explored the effects of *Bacillus* on lipid metabolism, and the limited results of these studies were simply obtained by detecting gene expressions and serum indexes (Do et al., 2015; Lei et al., 2015). Besides, it is worth noting that the lowered lipid accumulation induced by *Bacillus* was found to be accompanied by the increase of antioxidation (Do et al., 2015; Lei et al., 2015). Our previous study implied that *Bacillus amyloliquefaciens* SC06 (SC06) markedly elevated the antioxidant capacity of porcine intestinal epithelial cells (Wang et al., 2017). As oxidative stress is an obvious phenomenon in obesity, we hypothesis that SC06 may also prevent obesity and associated liver injury by regulating the antioxidant capacity and gut microbiota of hosts. In this study, we assessed the preventive effects of SC06 on HFD-induced obesity, liver injury and oxidative stress in mice and analyzed the intestinal microbiota structure.

## Materials and Methods

### Bacteria

*Bacillus amyloliquefaciens* SC06 (SC06) cells were stored in China Center for Type Culture Collection (No. M 2012280). The culture and preparation of SC06 was referred to previous study (Wang et al., 2017). Briefly, SC06 powder (10^8^ cfu/g) was prepared by Microbiology and Genetic Engineering Laboratory, Institute of Feed Sciences, Zhejiang University, China). SC06 was cultured on Luria-Bertani media, kept at 37°C for 24 h and shaken at 180 r/min. Pure bacterial cells were collected after centrifugation at 5000 *g* for 10 min at 4°C. Then, these cells were washed twice with sterile 0.85% sodium chloride solution. Ultimately, the culture purity and identification were constantly checked by the spreading plate method (Nikoskelainen et al., 2003).

### Animals and Diets

The experimental procedure was illustrated in Supplementary Figure S1. Sixty male C57BL/6J mice (6 weeks old, *n* = 15 per group) were obtained from Slac Laboratory Animal Co., Ltd. (Shanghai, China) and fed on normal chow diet for 1 week to adapt to the environment. Thereafter, animals were divided into four groups and fed with normal chow (NC group, 3616 Kcal/Kg energy), NC supplemented with 0.1% (w/w) SC06 powder (NC+SC06 group), HFD (HFD group, 80% NC, 0.5% cholesterol, 6.3% lard, 13% dried egg yolk, and 0.2% cholate, 4270 Kcal/Kg energy) and HFD supplemented with 0.1% (w/w) SC06 powder (HFD+SC06 group) for 8 weeks. During the preparation of the SC06 powder, starch was used to dilute SC06 and the same amount of starch was also added to the NC and HFD groups to compensate for the difference in nutrient composition of the diets. Normal chow diet was purchased from Xietong Organism Co., Ltd. (Nanjing, China). The nutritional constitutes of HFD was based on previous study (Xin et al., 2014). NC+SC06, HFD, and HFD+SC06 diets were all prepared by Xietong Organism Co., Ltd. (Nanjing, China). Mice were housed in standard plastic cages (three mice per cage) and maintained under a 12-h light-dark cycle at constant temperature and humidity [(23 ± 1)°C and (55 ± 5)%, respectively]. Mice body weight and food intake were recorded. The mass of white fat, including the perirenal fat, subcutaneous fat and epididymal fat was weighed. The experiment was approved by and performed in accordance with the guidelines of the ethics committee of Zhejiang University.

### Insulin Sensitivity

Oral glucose tolerance test (OGTT) and insulin tolerance test (ITT) were performed at the 7th week and 8th week, respectively. Before OGTT test, mice were fasted 8 h and were then given 2 g/Kg glucose orally. Blood glucose levels were determined with an Accu-chek glucose meter (Roche Diagnostics, Almere, Netherlands) at 0, 15, 30, 60, and 120 min. Before the ITT test, mice were fasted 4 h and insulin (0.75 U/kg) was injected intraperitoneally. Blood glucose levels were determined with an Accu-chek glucose meter (Roche Diagnostics, Almere, The Netherlands) at 0, 15, 30, 60, and 120 min.

### Western-Blotting Analysis

Liver tissues were resuspended in lysis buffer (Biotime Biotechnology, China), ground and rocked for 30 min on ice. Crude lysates were then centrifuged at 12000 rpm for 10 min. Western-blotting analysis was performed according to previous study (Wang et al., 2017).

### Biochemical Evaluation of Serum

The activities of glutamic-oxalacetic transaminase (GOT), glutamic-pyruvic transaminase (GPT), total-antioxidant capacity (T-AOC), superoxidase (SOD), and catalase (CAT) in serum were determined using commercial kits (Nanjing Jiancheng Bioengineering Institute, China) according to the instructions of the manufacturer. Interleukin (IL)-6, IL-1β, tumor necrosis factor (TNF)-α, and leptin concentrations were detected using a commercial ELISA kit (Raybiotech, GA, United States) according to the instructions of the manufacturer.

### Biochemical Evaluation of Liver

T-AOC, CAT activities as well as malonaldehyde (MDA), GSH levels in livers were determined using commercial kits (Nanjing Jiancheng Bioengineering Institute, China) according to the instructions of the manufacturer.

### Hematoxylin and Eosin, and Oil Red O Staining

Hematoxylin and eosin staining were performed according to previous study (Xin et al., 2014). For oil red O staining, 4-μm sections were cut from frozen optimal-cutting-temperature samples, affixed to microscope slides, and allowed to air-dry overnight at room temperature. The tissue sections were stained in fresh oil red O for 10 min and rinsed in water.

### Reactive Oxygen Species (ROS) Detection

Frozen liver sections were incubated with 5 mol/L Dihydroethidium (Sigma-Aldrich, United States) for 30 min (37°C). After washes with PBS, the ROS level in the tissue was measured by confocal microscopy (Leica, Germany).

### TUNEL Staining

The cell apoptosis in the liver was determined by TUNEL according to the manufacturer’s instructions In Situ Cell Death Detection Kit (KeyGen BioTECH, China).

### Transmission Electron Microscopy (TEM)

Liver tissue was harvested after perfusion with PBS, then fixed and processed as described previously (Stolz et al., 1999). After dehydration, thin sections were stained with uranyl acetate and lead citrate for observation under a 1024 × 1024 pixel CCD camera system (AMT Corp., United States).

### Fecal DNA Extraction and Illumina Miseq Sequencing

Genomic DNA was extracted using a TIANamp Stool DNA Kit according to manufacturer’s protocols (Tiangen Biotech, China). The V4 hypervariable region of 16S rRNA gene was amplified using the primer pair 515F (5′-GTGCCAGCMGCCGCGGTAA-3′) and 806R (5′-GGACTACHVGGGTWTCTAAT-3′), and MiSeq sequencing were performed by RiboBio Co., Ltd. (Guangzhou, China). All the DNA datasets have been submitted to the NCBI Sequence Read Archive database under the accession number SRP128678.

### Statistical Analysis

The data are presented as the means ± SD with respect to the number of samples (n) in each group. Statistical significance between multiple treatment groups was determined using analysis of variance (ANOVA). Tukey’s method was used to perform multiple comparison of means. *P*-value less than 0.05 was considered as statistical significance.

## Results

### SC06 Treatment Attenuated Obesity and Insulin Resistance

**FIGURE 1:**
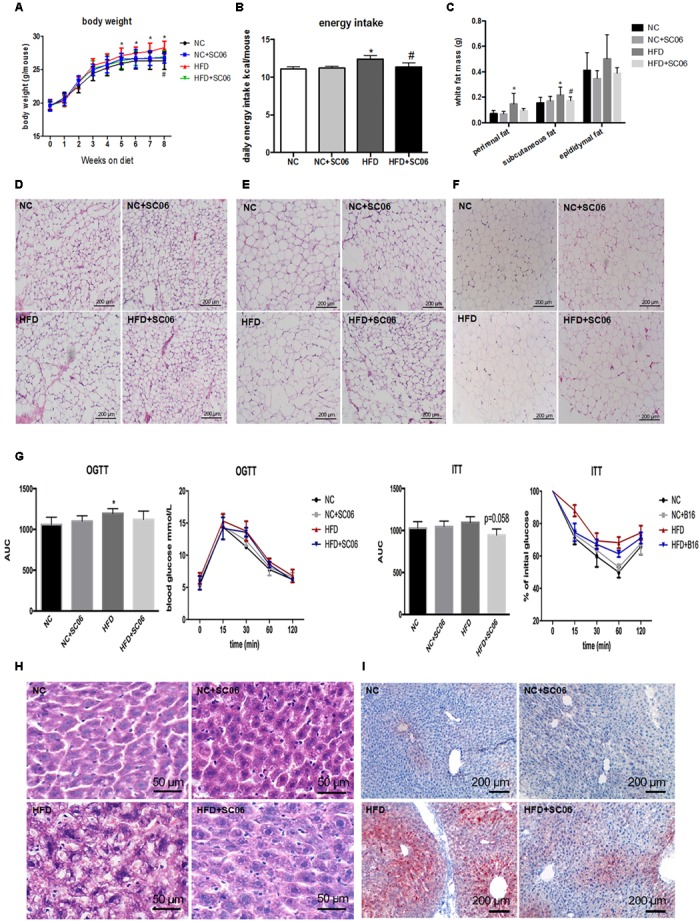
SC06 attenuated HFD-induced obesity, insulin resistance and hepatic steatosis. **(A)** Body weight, **(B)** daily energy intake, **(C)** partial white fat mass, **(D)** subcutaneous adipocyte morphology, **(E)** perirenal adipocyte morphology, **(F)** epididymal adipocyte morphology, **(G)**, OGTT and ITT, AUC means area under the curve. **(H)** HE liver sections, **(I)** oil-red O liver section. Results are expressed as means ± SD (*n* = 15 for body weight, energy intake, and fat mass; *n* = 4 for tissue morphology). Differences between groups were determined by one-way ANOVA followed by Tukey’s *t*-test. Significant differences between HFD versus NC are indicated as ^∗^*p* < 0.05. Significant differences between HFD versus HFD+SC06 are indicated as ^#^*p* < 0.05.

Compared with NC, NC+SC06 did not alter body weight significantly. HFD-fed mice gained more weight from week 5 than NC-fed mice (*p* < 0.05), but HFD+SC06 significantly relieved the HFD-induced body weight increases (*p* < 0.05, Figure 1A). Besides, the daily energy intake of mice in HFD group was increased compared with NC group (*p* < 0.05), while HFD+SC06 decreased energy intake significantly (*p* < 0.05, Figure 1B). Perirenal and subcutaneous partial fat weights were not changed in NC+SC06-fed mice compared to NC-fed mice, but were higher in mice receiving HFD (*p* < 0.05). Although weights of the perirenal, subcutaneous and epididymal fat were consistently decreased in HFD-fed mice received SC06, only subcutaneous fat weight reduced significantly (*p* < 0.05, Figure 1C). Furthermore, histological analysis of perirenal, subcutaneous and epididymal adipose tissues showed that the size of adipocytes in the HFD group was greater than that in NC, but the size of adipocytes in the HFD+SC06 group was less than that in HFD group (Figure 1D–F). In addition, HFD significantly increased glucose levels during the OGTT (*p* < 0.05, Figure 1G), and HFD+SC06 elevated the clearance of glucose slightly. Moreover, HFD worsened the insulin resistance slightly, while HFD+SC06 marginally improved the insulin sensitivity (*p* = 0.058).

### SC06 Treatment Alleviated Liver Steatosis and Injury of HFD-Induced Mice

**FIGURE 2:**
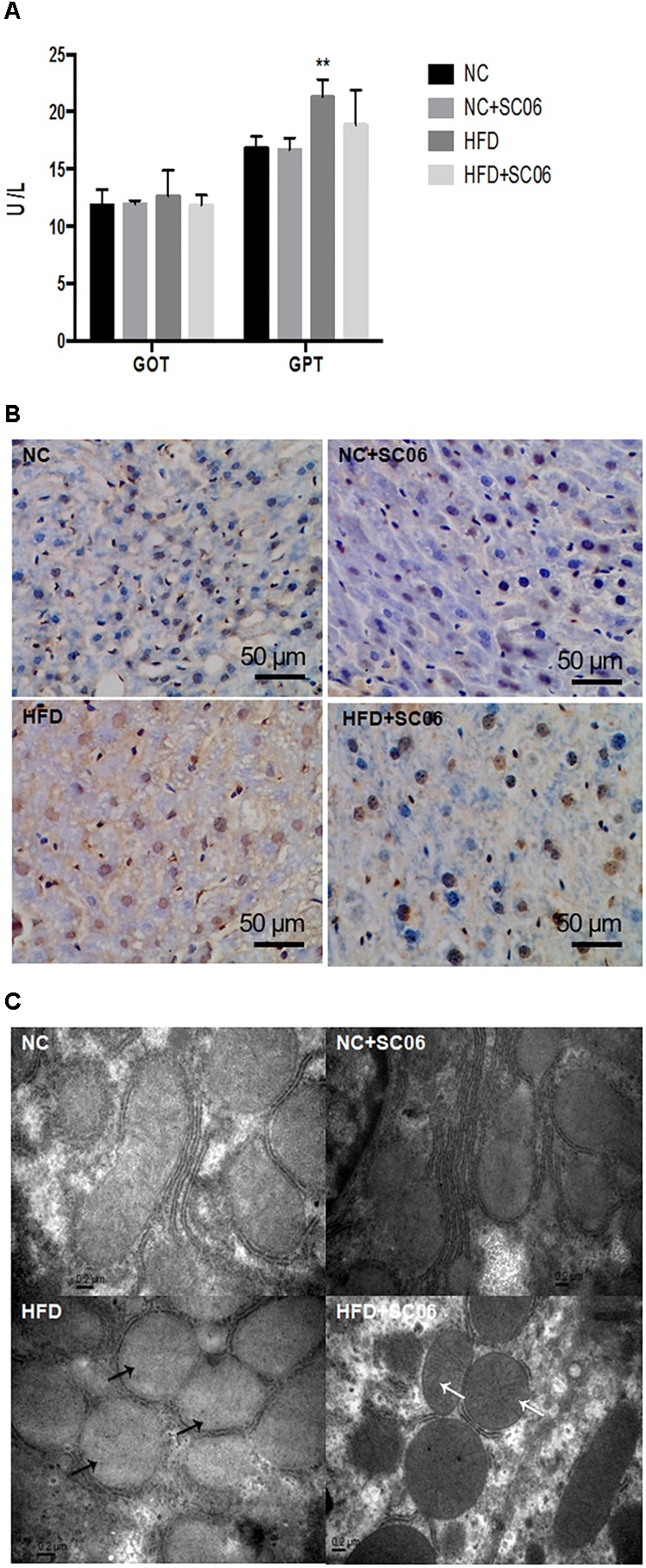
SC06 attenuated liver lesion. **(A)** GOT and GPT activities, **(B)** TUNEL sections, **(C)** electron microscopic sections. The black arrows indicate the abnormal mitochondria, including the disappearance of cristae and swelling. The white arrows indicate the normal mitochondrial. Results are expressed as means ± SD. Differences between groups were determined by one-way ANOVA followed by Tukey’s *t*-test (*n* = 6 for GOT and GPT; *n* = 4 for TUNEL and TEM). Significant differences between HFD versus ND are indicated as ^∗∗^*p* < 0.01.

The HE and oil-red O liver sections from HFD mice exhibited visible intracellular vacuolization and marked lipid accumulation. Compared with the HFD group, the degree of hepatic steatosis was appreciably decreased by SC06 treatment (Figure 1H,I). The results of GOT and GPT activities showed that HFD or/and SC06 had no significant effect on GOT activity. However, GPT activity was significantly enhanced in HFD group (*p* < 0.01), but decreased slightly in HFD+SC06 (Figure 2A). Additionally, TUNEL results indicated that HFD enhanced liver cell apoptosis, which was reduced by SC06 treatment (Figure 2B). The mitochondrial TEM revealed that HFD seriously induced mitochondrial abnormalities, including swelling and disappearance of cristae, which were effectively alleviated by administration SC06 (Figure 2C).

### SC06 Treatment Decreased the Secretion of Inflammatory Factors and Adipokine of HFD-Induced Mice

**FIGURE 3:**
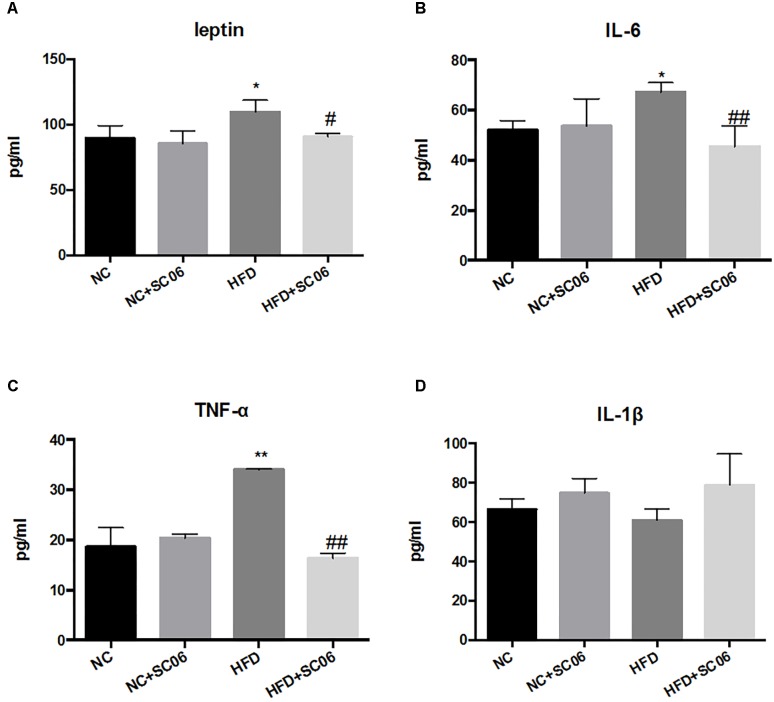
SC06 modulated secretion of inflammatory factors and adipokine. **(A)** Leptin, **(B)** IL-6, **(C)** TNF-α, **(D)** IL-1β. Results are expressed as means ± SD. Differences between groups were determined by one-way ANOVA followed by Tukey’s *t*-test (*n* = 6). Significant differences between HFD versus NC are indicated as ^∗^*p* < 0.05, ^∗∗^*p* < 0.01. Significant differences between HFD versus HFD+SC06 are indicated as ^#^*p* < 0.05, ^##^*p* < 0.01.

No significant changes were found for serum levels of IL-6, IL-1β, TNF-α and leptin between NC and NC+SC06 groups (Figure 3A–D). As expected, leptin, IL-6 as well as TNF-α levels were significantly elevated in HFD group (*p* < 0.05), but lowered in HFD+SC06 (Figure 3A–C) (*p* < 0.05). However, IL-1β level was not altered significantly in HFD and/or HFD+SC06 groups (Figure 3D).

### SC06 Relieved Liver Oxidative Stress of HFD-Induced Mice

Table 1 shows that mice fed with NC+SC06 exhibited higher hepatic CAT activity (*p* < 0.05). Whereas, NC+SC06 had no significant effects on MDA, T-AOC, GSH levels and SOD activity in liver. HFD significantly increased MDA content (*p* < 0.05), which was lowered significantly by HFD+SC06 (*p* < 0.05).

**Table 1:** HFD and SC06 effects on liver antioxidant parameters.

Parameters	NC	NC+SC06	HFD	HFD+SC06
MDA (nmol/mg prot)	2.66 ± 0.22	2.57 ± 0.13	3.59 ± 0.17^∗^	2.36 ± 0.05^#^
T-AOC (U/mg prot)	0.49 ± 0.01	0.56 ± 0.12	0.46 ± 0.13	0.57 ± 0.04
GSH (mg/g prot)	1.55 ± 0.32	1.52 ± 0.32	1.45 ± 0.31	1.23 ± 0.23
CAT (U/mg prot)	13.84 ± 1.73	17.06 ± 2.14^∗^	15.27 ± 1.27	16.66 ± 1.71
SOD (U/mg prot)	141.67 ± 27.59	135 ± 12.42	157.03 ± 26.61	166.52 ± 21.89

*Results are expressed as means ± SD. Differences between groups were determined by one-way ANOVA followed by Tukey’s t-test (n = 6). Significant differences between HFD versus NC are indicated as ^∗^p < 0.05. Significant differences between HFD versus HFD+SC06 are indicated as ^#^p < 0.05.*

**FIGURE 4:**
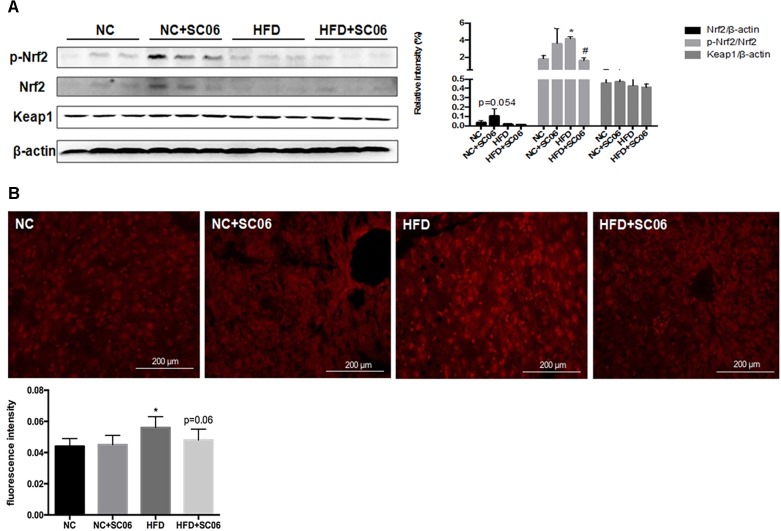
SC06 regulated Nrf2/Keap1 signaling pathway and ROS level. **(A)** Total protein levels of Keap1 and β-actin as well as the phosphorylated and total protein levels of Nrf2 in mice livers were determined using Abs recognizing phospho-specific or total protein, **(B)** ROS frozen sections. Fluorescence intensity was quantified by ImageJ software. Results are expressed as means ± SD. Differences between groups were determined by one-way ANOVA followed by Tukey’s *t*-test (*n* = 6). Significant differences between HFD versus ND are indicated as ^∗^*p* < 0.05. Significant differences between HFD versus HFD+SC06 are indicated as ^#^*p* < 0.05.

Nrf2-Keap1 is a well-characterized pathway that responds to oxidative stress and regulates genes of phase II detoxifying enzymes through binding with antioxidant-responsive element (Turnbaugh et al., 2008; Jones et al., 2015). Figure 4A shows that Nrf2 expression was marginally increased in mice feeding NC+SC06 (*p* = 0.054). Meanwhile, the phosphorylation of Nrf2 was significantly up-regulated in HFD group compared to NC (*p* < 0.05). Compared to HFD, mice receiving HFD+SC06 showed down-regulated p-Nrf2 expression (*p* < 0.05). No significant differences were observed in Keap1 expressions among all groups (Figure 4A).

Oxidative stress is derived either from an increase in ROS production or decreased levels of ROS scavenging. According to Figure 4B, HFD elevated ROS levels in liver (*p* < 0.05), but HFD+SC06 inhibited the elevated ROS concentration induced by HFD, although this was not statistically significant (*p* = 0.06).

### Overall Structural Modulation of Gut Microbiota After SC06 Treatment

**FIGURE 5:**
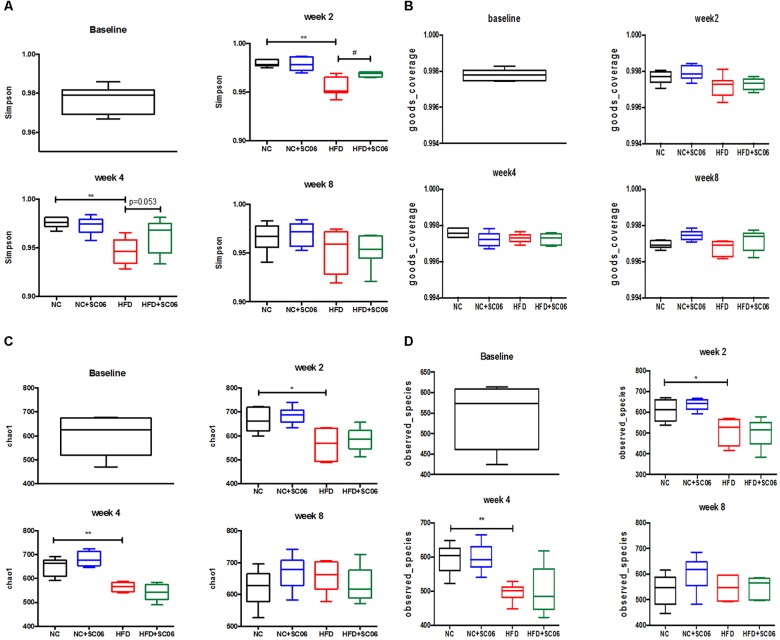
Changes in a-diversity of gut microbiota communities, as measured by **(A)** Simpson’s, **(B)** Goods_coverage’s, **(C)** Chao1’s, and **(D)** Observed_species’ Index Calculator. Differences between groups were determined by one-way ANOVA followed by Tukey’s *t*-test (*n* = 5). Significant differences between HFD versus NC are indicated as ^∗^*p* < 0.05, ^∗∗^*p* < 0.01. Significant differences between HFD versus HFD+SC06 are indicated as ^#^*p* < 0.05.

α-diversity (richness and evenness) of the communities was measured by Simpson’s, Goods_coverage’s, Chao1’s and Observed_species’ indexes, respectively (Figure 5). After 2- and 4-week HFD feeding, α-diversity was much higher than that of NC (*p* < 0.05). However, at the 8th week, there was no difference between the two groups (Figure 5A,B,D). In addition, as shown by the Simpson’s index, we observed that HFD+SC06 increased the α-diversity obviously at the 2nd week (*p* < 0.05) and the 4th week (*p* = 0.053), but not at the 8th week (Figure 5A). Principal component analysis revealed that gut microbiota in HFD group deviated from NC structure. At the 8th week, the gut bacterial composition profile in HFD+SC06 changed toward control group (Supplementary Figure S2).

**FIGURE 6:**
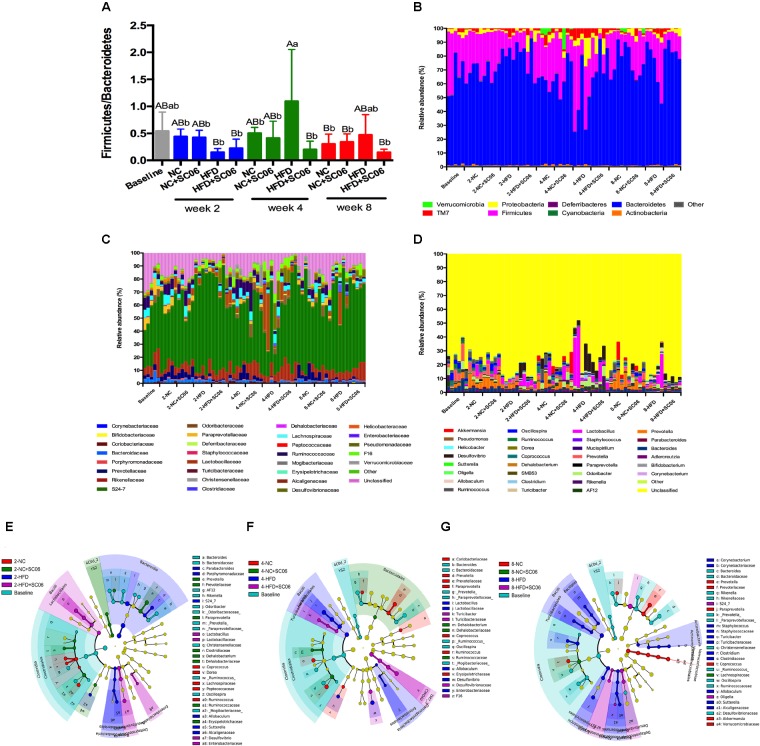
SC06 administration changed gut microbiota. **(A)** The ratio between Firmicutes and Bacteroidetes of gut microbiota, **(B)** phyla, **(C)** families, **(D)** genus of mice. Cladogram plotted from LeFSe analysis showing the taxonomic levels represented by rings with phyla in the outermost the ring and genera in the innermost ring. Each circle is a member within that level. **(E)** Week 2, **(F)** week 4, and **(G)** week 8.

Histograms illustrating the gut microbiota structure revealed the microbial species and their relative abundance (Figure 6). As obesity is characterized by a decrease in Bacteroidetes and an increase of Firmicutes (Nicholson et al., 2012). In the present study, we first determined the ratio of Firmicutes/Bacteroidetes. At the 4th week, HFD was associated with a significant increase in Firmicutes/Bacteroidetes (*p* < 0.05). Moreover, HFD+SC06 lowered Firmicutes/Bacteroidetes obviously compared to HFD-fed mice (Figure 6A). At phylum level, we also noticed that HFD increased Proteobacteria and *TM7*, especially at the 4th week (*p* < 0.05), while HFD+SC06 reduced TM7 abundance at the 4th week (*p* = 0.06). On the contrary, Verrucomicrobia almost disappeared in both HFD and HFD+SC06 groups (Figure 6B,C and Supplementary Figure S3). At family level, we found that *S24-7* family accounted for the majority. At the 4th and 8th weeks, Clostridiaceae relative abundance was increased by HFD but inhibited by HFD+SC06 (*p* < 0.05) (Figure 6C and Supplementary Figure S3B). Furthermore, although most genera were unclassified, we also found that the genera profiles in HFD groups were distinct from that of NC groups. It is worth noting that increased *Prevotella* in HFD group was inhibited by HFD+SC06 at the 2nd week (*p* < 0.05) (Figure 6D and Supplementary Figure S3C). To compare community characteristics in more detail, heatmaps were constructed representing the relative abundance of species (Supplementary Figure S4). Results showed that most members of the gut microbiota belonging to *S24-7* genus. The altered species in HFD group were seldom returned to normal by SC06 treatment at the 2nd and 4th weeks. However, some of the decreased *S24-7* species (*S24-7*_346870, 315430, 264734, etc.) in mice receiving HFD were reversed in HFD+SC06 group at the 8th week.

LeFSe depicted time course changes of the dominant microbiota among groups (Figure 6E–G). We observed that at the 2nd week, the predominant intestinal flora in HFD were Bacteroidia and Betaproteobacteria. However, as time went on, branches of *Bacilli* became the major ones in HFD group at both 4th and 8th weeks. With HFD+SC06 feeding for different time points, the branches of *Desulfovibrio*, *S24-7* and *F16* were identified as novel superior microbiota (Figure 6).

## Discussion

Consistent with previous research (Xin et al., 2014), here we found that using the same HFD also successfully induced mice obesity and the related metabolic disorder, indicated by higher fat deposition, insulin resistance and hepatic steatosis. However, SC06 treatment effectively improved these features of metabolic syndrome. Coelomate animals coevolve with a diverse range of gut microbiota (Nicholson et al., 2012). The coevolution between host and gut microorganisms have led to a mutualistic relationship in which the microbiota contributes to many host physiological processes and in turn, hosts provide niches and nutrients for microbial survival (Kamada et al., 2013). When the mutualistic relationship is disrupted, the disordered gut microbiota leads to diseases (Littman and Pamer, 2011). Obesity and associated metabolic disorders are characterized by “low-grade” inflammation (Hotamisligil, 2006). Changes in gut microbiota and epithelial functions may play a crucial role in inflammation associated with obesity (Cani et al., 2008). In this study, with the reduction of obesity, HFD+SC06 also significantly decreased the concentration of IL-6 and TNF-α compared to that of HFD. Thus, the role of SC06 in improving obesity and inflammation promoted us to figure out whether SC06 also attenuated gut bacterial dysbiosis. In mouse, the major distal gut microbiota are members belonging to Bacteroidetes and Firmicutes (Ley et al., 2005). Many studies have shown that the increased ratio of Firmicutes and Bacteroidetes is associated with the obesity phenotype (Ley et al., 2005; Moschen et al., 2012). In agreement with these findings, we observed that compared to NC group, Firmicutes/Bacteroidetes in HFD increased about twofold, which was decreased efficiently by SC06 administration. We also noticed that HFD increased relative abundance of *TM7* and Proteobacteria at the 4th week, but decreased Verrucomicrobia and enriched *TM7* was suppressed by SC06. In a previous report, obese individuals contained lower Verrucomicrobia abundance as well (Clarke et al., 2012). It was also suggested that *Nlrp6*-deficient mice with overrepresentation of Prevotellaceae and *TM7* increased susceptibility to liver injury when on a methionine-choline-deficient to induce NASH (Elinav et al., 2011). *TM7* can induce changes that result in inflammation in the colon and adipose tissues as well (Henao-Mejia et al., 2012). Differences were found not only at phyla level but also at all lower taxa levels among groups. Although it is shown that Clostridiaceae families were lowered in diet-induced obese mice (Clarke et al., 2013), a recent study found that Clostridiaceae was enriched in NAFLD patients (Jiang W.W. et al., 2015). Here, we found the increased Clostridiaceae induced by HFD was suppressed by SC06 treatment. Additionally, we also identified bacterial species whose abundance were altered by HFD and barely returned to control levels with SC06 approaches at the 2nd and 4th weeks. However, the abundance of species belonging to *S24-7*, including *S24-7*_346870, 315430 and 264734, was reversed by probiotic treatment at the 8th week. Bacteroidales *S24-7* is a butyrate-producing bacterium associated with intestinal epithelial cell health (Villanueva-Millan et al., 2015). Qiu et al. (2017) believed *S24-7* is negatively correlated with trimethylamine N-oxide, which can enhance accumulation of macrophage cholesterol and foam cell formation. On the contrary, Wu et al. (2017) considered *S24-7* as the bad gut microbiota. Based on our results, we speculate that the increase of specific *S24-7* strains might exert beneficial effect on the host. However, we didn’t notice significant differences for *S24-7* abundance at family level between HFD and HFD+SC06 groups.

Because of the anatomical position and its unique vascular system, the liver is susceptible to the microbial products from the gut (Seo and Shah, 2012). Derangement of the gut microbiota occurs in a large percentage of patients with chronic liver disease, including NAFLD and NASH (Compare et al., 2012). The close interplay exists between the gut and liver is named “gut-liver axis.” Here, we also examined the liver injury in obese mice. HFD obviously increased GPT activity in the serum and elevated lipid accumulation, apoptosis and mitochondrial injury in the liver, which were reversed effectively in HFD+SC06. Lipid peroxidation and secondary cellular injury are the dominant mechanism in the transition from relatively stable hepatic steatosis to potentially progressive steatohepatitis in NAFLD. Oxidation of excessive fatty acids generates ROS that damage organelles and stimulate signaling pathways leading to fibrosis and cellular injury (Chang et al., 2006). Through analyzing the hepatic oxidation, we found that NC+SC06 significantly increased liver CAT activity. What’s more, oxidative stress was induced by HFD, but inhibited by SC06 administration, which was represented by the decreased hepatic ROS level and MDA concentration. Moreover, Nrf2 is a transcriptional activator that regulates cytoprotective gene expressions (Ishii et al., 2000). During oxidative stress, Nrf2 is released from its cytosolic repressor Keap1. The phosphorylated Nrf2 translocates into the nucleus where it binds to antioxidant reaction element residing within the promoter regions of many antioxidant and phase II genes (Lee and Johnson, 2004). According to our results, NC+SC06 elevated Nrf2 level to enhance the antioxidant ability. As a stress factor, HFD also significantly increased the phosphorylation of Nrf2 to resist the oxidative stress induced by diet. In the previous study, compounds with antioxidant capacity could activate Nrf2 pathway in combination with HFD (Feng et al., 2017). However, in the present study, with HFD+SC06 treatment, p-Nrf2 expression was markedly decreased. As far as our concerned, since the ROS and MDA levels were reduced in HFD+SC06 treated mice, it is not necessary for hepatocytes to activate more Nrf2 and increase antioxidase activities to combat with oxidative stress. Additionally, recent studies demonstrated Nrf2 ablation in mice adipose tissue prevented diet-induced obesity (Zhang et al., 2016), and mice lacking Nrf2 had more energy expenditure and resisted to diet-induced obesity (Schneider et al., 2016). Thus, as far as we concerned, the obvious inactivation of Nrf2 induced by HFD+SC06 might also protect against HFD-induced lipid accumulation.

**FIGURE 7:**
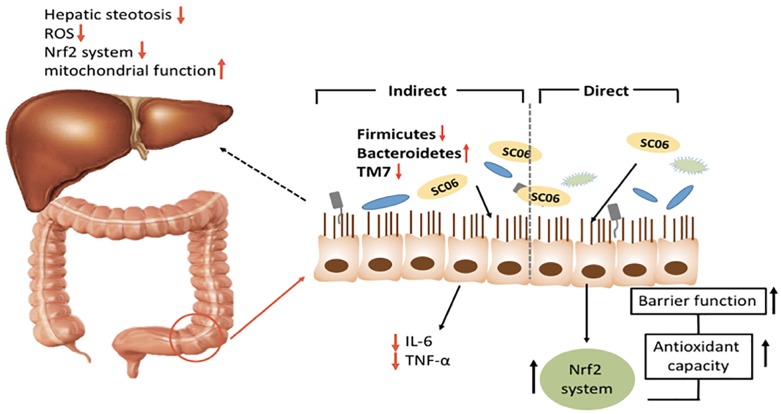
Graphical summary of multiple roles of SC06 in improving HFD-induced obesity and liver injury.

Epithelial function is important for the development of NAFLD and obesity (Raybould, 2012). In a previous research, we suggested that SC06 was able to elevate the oxidation resistant capacity of intestinal porcine epithelial cell line (Wang et al., 2017), and the present study has provided novel information regarding the effects of SC06 on gut microbiota, hepatic steatosis and oxidative stress in mice (Figure 7).

Taken together, we propose that SC06 protected mice from HFD-induced obesity and liver injury through (1) directly improving the epithelial function by enhancing intestinal epithelial cell antioxidant capacity, (2) decreasing inflammation, lowering hepatic oxidative damage and (3) regulating gut microbiota composition, especially Bacteroides, Firmicutes, *TM7* phyla and species belonging to *S24-7*. These findings may aid in the application of probiotic *Bacillus* in the food industry to improve human and animal’s health. However, further investigation about the correlations among the decreased obesity, oxidative stress and gut bacterial composition are needed.

## Author Contributions

WL and YPW designed the experiments. YPW, HX, and XX performed the animal husbandry. BW, XZ, and JN analyzed the 16S rRNA data. YW and XM did the animal experiments. YW wrote the final manuscript. All authors approved and contributed to the final version of the manuscript.

## Conflict of Interest Statement

The authors declare that the research was conducted in the absence of any commercial or financial relationships that could be construed as a potential conflict of interest.
