# Supplementary material for: Bacillus amyloliquefaciens SC06 Protects Mice Against High-Fat Diet-Induced Obesity and Liver Injury via Regulating Host Metabolism and Gut Microbiota
Source: Front Microbiol. 2019 May 28;10:1161. doi: 10.3389/fmicb.2019.01161 (PMC6547872; doi:10.3389/fmicb.2019.01161)
Supplement: Supplementary file 1 [file Table_1.DOCX]

Supplementary Material

***Bacillus amyloliquefaciens* SC06 Protects Mice Against High-Fat Diet-Induced Obesity and Liver Injury via Regulating Host Metabolism and Gut Microbiota**

Yang Wang^1,2^, Yanping Wu^1^, Baikui Wang^1^, Han Xu^1^, Xiaoqiang Mei^1^, Xiaogang Xu^1^, Xiaoping Zhang^3*^, Jiajia Ni^4,5*^, Weifen Li^1*^

^1^Key Laboratory of Molecular Animal Nutrition of the Ministry of Education, Institute of Feed Science, College of Animal Sciences, Zhejiang University, Hangzhou 310058, China

^2^College of Animal Science and Technology, Qingdao Agricultural University, Qingdao, 266109, China

^3^China National Bamboo Research Center, Key Laboratory of High Efficient Processing of Bamboo of Zhejiang Province, Hangzhou, Zhejiang 310012, China

^4^Department of Hepatobiliary Surgery II, Guangdong Provincial Research Center of Artificial Organ and Tissue Engineering, Zhujiang Hospital of Southern Medical University, Guangzhou, 510280, China

^5^State Key Laboratory of Organ Failure Research, Southern Medical University, Guangzhou, 510515, China

*** Correspondence:**^1^Weifen Li
[weifenli@zju.edu.cn](mailto:weifenli@zju.edu.cn)

^2^Jiajia Ni

[nijiajia@126.com](mailto:nijiajia@126.com)

^3^Xiaoping Zhang

[zhangxiaoping403@gmail.com](mailto:zhangxiaoping403@gmail.com)


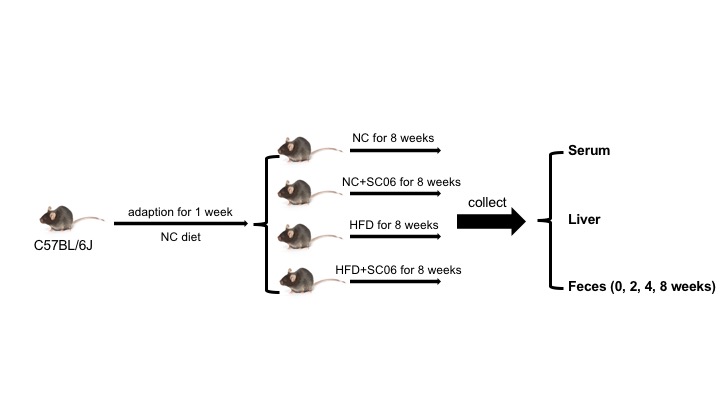
**Supplementary Figure 1.** Graphical summary of the experimental procedure


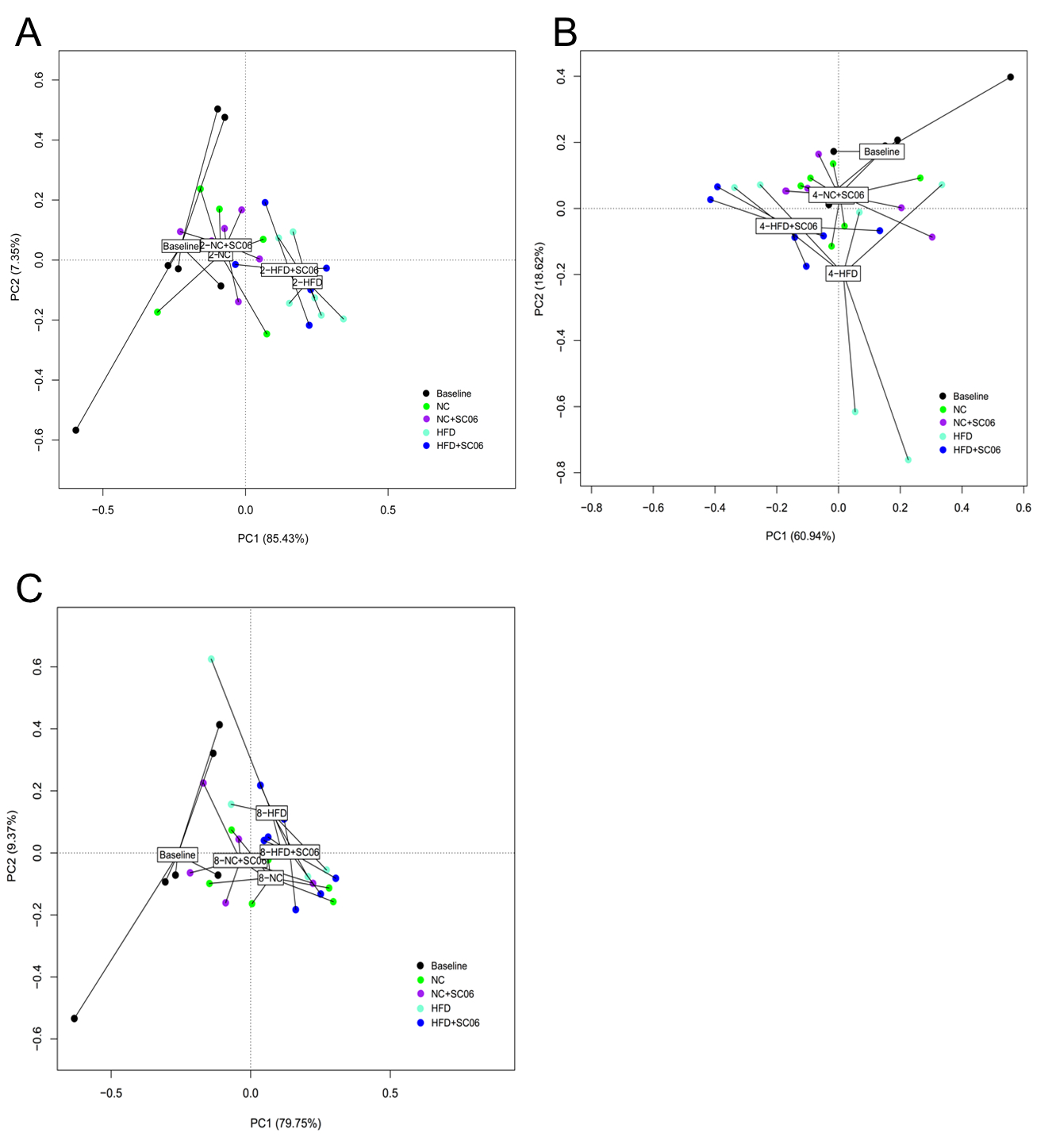


**Supplementary Figure 2.** Principal component analysis at genus level, (**A**) week 2, (**B**) week 4, (**C**) week 8. ●Baseline ●NC●NC+SC06 ●HFD●HFD+SC06


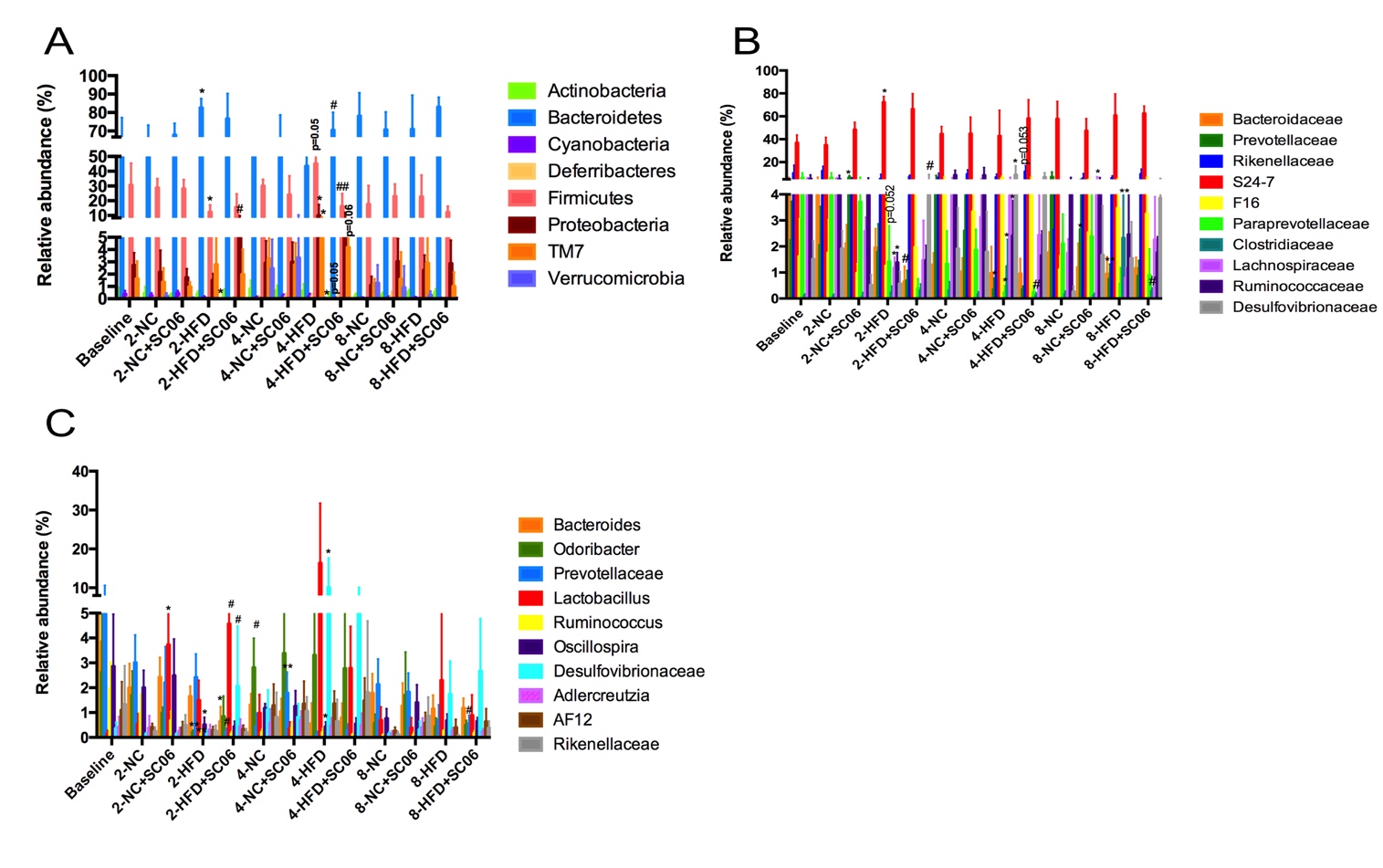


**Supplementary Figure 3.** Relative abundance of gut microbiota at different taxa levels (**A**) phyla, (**B**) family, (**C**) genera. Differences between groups were determined by one-way ANOVA followed by Tukey’s *t*-test. Significant differences between HFD versus NC in the same week are indicated as ^*^*p* < 0.05, ^**^*p* < 0.01. Significant differences between HFD versus HFD+SC06 in the same week are indicated as ^#^*p* < 0.05, ^##^*p* < 0.01.

A

B

C

**Supplementary Figure 4.** Heatmaps of the relative abundance of dominant microbial species altered by HFD or/and SC06 treatment at (**A**) week 2, (**B**) week 4, and (**C**) week 8.
